# Effects of adverse childhood health experiences on cognitive function in Chinese middle-aged and older adults: mediating role of depression

**DOI:** 10.1186/s12889-023-16169-7

**Published:** 2023-07-05

**Authors:** Gaoling Wang, Yuqin Zhou, Jing Duan, Qianqian Kan, Zhaopeng Cheng, Shaoliang Tang

**Affiliations:** School of Health Economics and Management, Nanjing University of Chinese Medicine, 138 Xianlin Road, Qixia District, Nanjing, China

**Keywords:** Adverse Childhood Health Experiences, Cognitive function, Depression, Middle-aged and older adults

## Abstract

**Background:**

Adverse childhood experiences are critical factors in depression and cognitive decrease, but the effect of adverse childhood health experiences (ACHEs) on cognitive function and the role of depression have not been fully studied.

**Methods:**

Data were taken from the China Health and Retirement Longitudinal Study (CHARLS) of 2014 and 2018. This study used indicators of situational memory ability and mental status to measure cognitive capacity. Besides analyzing the different types of ACHEs, scores for ACHEs were calculated to represent the severity of ACHEs. The Center for Epidemiologic Studies Depression Scale (CES-D) was used to assess depression. The analysis of this study employed two different analytical strategies in order to examine the mediated effects of depression. We used Sobel’s test and Baron and Kenny’s causal step approach, which utilized a generalized least squares regression model. Furthermore, a logistic regression model was used to evaluate the robustness of the Karlson-Holm-Breen (KHB) approach.

**Results:**

In this study, 6301 individuals who met the requirements of the study were included. We found that being confined to bed (ACHE3) (β=-0.3846, p = 0.022) in childhood had a negative impact on cognitive function. Similarly, ACHEs had a negative effect on cognitive function (β=-0.0819, p = 0.090). And after the depression had been introduced into the model, the regression coefficient of ACHEs on cognitive function was no longer significant (β=-0.0170, p = 0.727). The Sobel test showed that for ACHE3, the mediated proportion of the total effect of depression was 36.92%. While for ACHEs, the proportion of the mediated effect of depression was 70.11%. Finally, a robustness test of the mediating effect using the KHB method revealed that the mediating effect still existed. Further, based on different gender, age, and educational levels, the heterogeneity test indicated that the relationship between ACHEs and cognitive function and mediating effects of the depression were different as well as passing the robustness test of the interaction.

**Conclusion:**

The decline in cognition had been shown to be correlated with ACHEs and depression mediated this relationship. Positive interventions might help to improve cognitive performance in individuals suffering from ACHEs and depression.

**Supplementary Information:**

The online version contains supplementary material available at 10.1186/s12889-023-16169-7.

## Introduction

The cognitive function of an individual is one of the most significant indicators of health [1]. Normal aging is accompanied by cognitive decline, and severe cognitive decline even results in mild cognitive impairment (MCI) [2]. As defined by Peterson in 1999, mild cognitive impairment (MCI) is a state that occurs between normal aging and dementia. It can be referred to as a transitional state between both types of disorders [3]. Indeed, the typical feature of the onset of dementia is MCI, which is one of the most prominent burdens of an aging population.

With the aging of the worldwide population, the number of people who suffer from dementia is predicted to increase to 82 million by 2030 and almost double by 2050 worldwide [4]. According to the data reported in the China Census Yearbook, China has 260 million people over 60 years old, accounting for 18.7% of the total population of 1.4 billion in 2019 [5]. Meanwhile, nearly 15.07 million people with dementia are aged 60 years and above in China, and this number accounts for approximately 25.5% of the entire population with dementia worldwide [6]. The number of people with dementia in China is already the highest in the world [7]. The study revealed that the incidence, mortality, and disability-adjusted life year (DALY) rates of dementia in the Chinese population in 2019 tended to increase with age and had increased by more than 150% each year for the past three decades [8].

A number of factors contribute to cognitive decline, including education level [9, 10], socioeconomic status (SES) [11, 12], and chronic illnesses, such as strokes [13] and diabetes [14]. Recently, a growing number of studies have begun to focus on early life experiences, which appear to be associated with dementia and cognitive impairment in later life [15]. Based on the life-course perspective, it is considered that early life experiences are of great significance. Actually, there is no denying that early exposure plays out in adulthood, and it does not need to be physical for these early experiences to have an impact on later life health [16, 17], and this implies that early life experiences are associated with psychological changes that have a lasting effect on health in later life. Also, due to the particular sensitivity of cognitive function during childhood to environmental factors, such changes can also have a lasting impact on the quality of life throughout life [18]. In addition, according to the cumulative advantage/disadvantage theory, risk exposure over the course of a lifetime is cumulative, and disadvantages experienced at different points in time may have an impact on later health problems [19]. Even so, the majority of existing research had focused on the effects of adverse childhood experiences (ACEs) which included childhood abuse, poverty, or dysfunctional family functioning on adult health. For example, researchers have demonstrated that any adversity suffered during childhood has been associated with a wide range of health outcomes in adulthood, such as depression [20–23], immune dysfunction [24], cardiovascular disease [25–28], and disturbances in sleep [29]. Several studies have examined adverse childhood health status as well, suggesting that childhood health status remains a significant factor in functional health trajectories in old age [30]. A study conducted from The China Health and Retirement Longitudinal Study (CHARLS) in 2011 confirmed the existence of a significant link between the health of Chinese children and their adult health. A study for 7 598 Chinese adults over 45 found that caring for their childhood was critical to their health later in life [31]. However, in our review, there were no studies that included adverse experiences due to health problems in adverse childhood experiences (ACEs) and much less evidence of the impact of these childhood experiences on cognitive health in adults. We have proposed that adverse childhood health experiences (ACHEs) stand for poor health condition and the adverse experiences that result from health condition in childhood based on the definition of ACEs which stands for multiple adverse events that happened in childhood [32, 33]. In our study, we thought that this concept might be applied to health-related adverse childhood experiences (ACEs). At the same time, we proposed the following question: Do ACHEs affect cognitive function in Chinese middle-aged and older people?

Additionally, a substantial amount of research has been conducted on the relationship between ACEs and depression, suggesting that ACEs are risk factors for depression [20, 34, 35]. According to the sociological perspective of social integration and mental health, social integration at the community and organizational levels contributes to positive mental health [36–38]. It is generally understood that social integration refers to the extent to which people are interconnected within a society or community group. The main source of social integration for children is friendship networks, and the vast majority of friendship networks are developed at school and maintained [39]. School-age children who are recovering from illness, or who have ACHEs, such as dropping out of school or being hospitalized, will be intermittently socialized throughout these experiences thereby failing to maintain friendship networks. According to our definition, ACHEs include poor health status during childhood. Whereas the consequences of poor health in children may accumulate over time and eventually result in chronic disease [40]. Meanwhile, children who suffer from pediatric or chronic illnesses are less likely to participate socially [41], thereby reducing their social integration. And it is clear from the evidence that there is an association between social integration during childhood and depression [42, 43]. As a result of what has been discussed above, it is reasonable to assume that there is a relationship between them.

Furthermore, it has been found that depression has been implicated as one of the risk factors for dementia, recent years have seen significant attention paid to depression because of its association with cognition [44], and significant correlations have been demonstrated in recent studies to suggest that depression is closely related to cognition [45–47]. Also, it is generally observed that depression plays a mediating role in studies of cognitive abilities [48–51]. Therefore, the purpose of this study was to investigate whether ACHEs have an impact on cognitive function in Chinese middle-aged and older adults through depression levels as a mediating variable.

It is important to note that the concept of cumulative risk has previously been used by researchers for measuring ACEs, such as constructing a scale to measure the number of adverse experiences and using the outcomes of the scale as predictors of future outcomes [52], however, the severity of ACEs was not taken into consideration when the study was conducted. As part of a study to explore the relationship between ACHEs and cognitive function in Chinese middle-aged and older adults, we included the degree of exposure to ACHEs. The exposure score can be used to measure how ACHEs affect cognitive function.

**Fig. 1:**
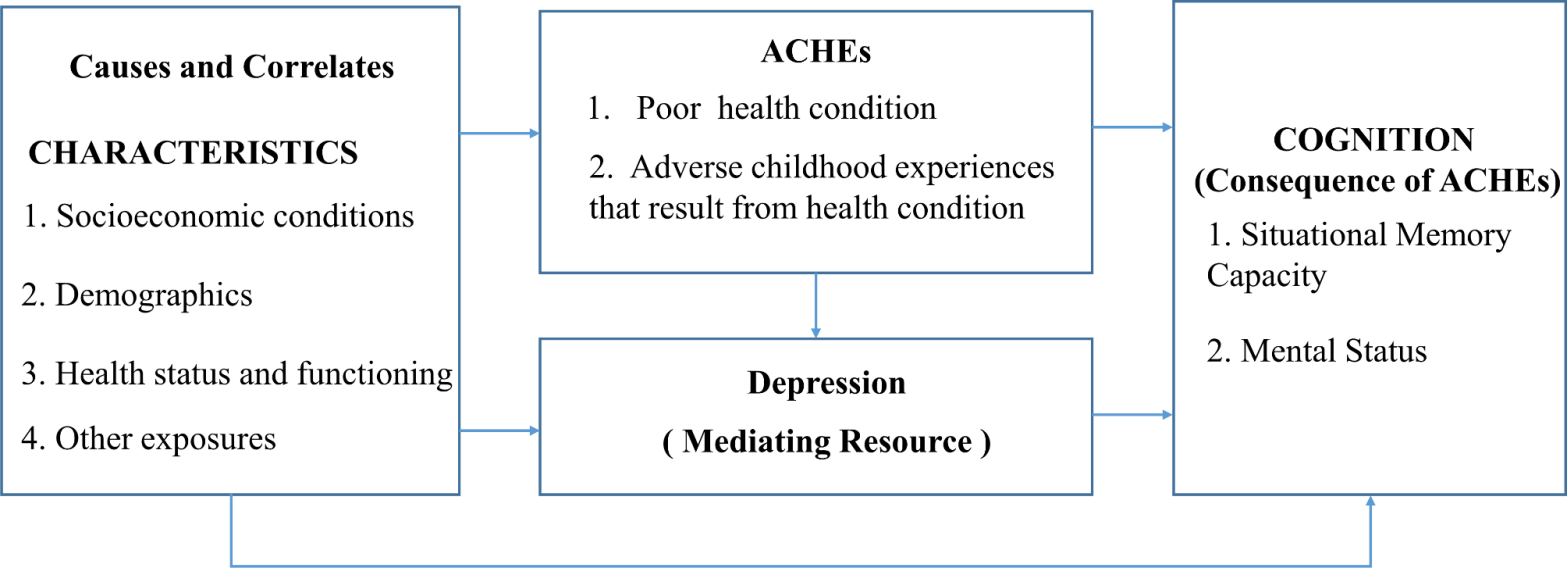
The proposed conceptual framework

According to the stress process model, it is critical to examine how stressors combine over the life course [53]. In our study, we proposed a conceptual framework (as shown in Fig. 1.) based on the literature and theories mentioned above and the stress process model. ACHEs were regarded as a primary stressor that might ultimately lead to adverse health outcomes (stress outcomes). Cognition was examined as a consequence of ACHEs in this study. Meanwhile, depression served as a mediator in the relationship between ACHEs and cognition.

## Materials and methods

### Study sample

**Fig. 2:**
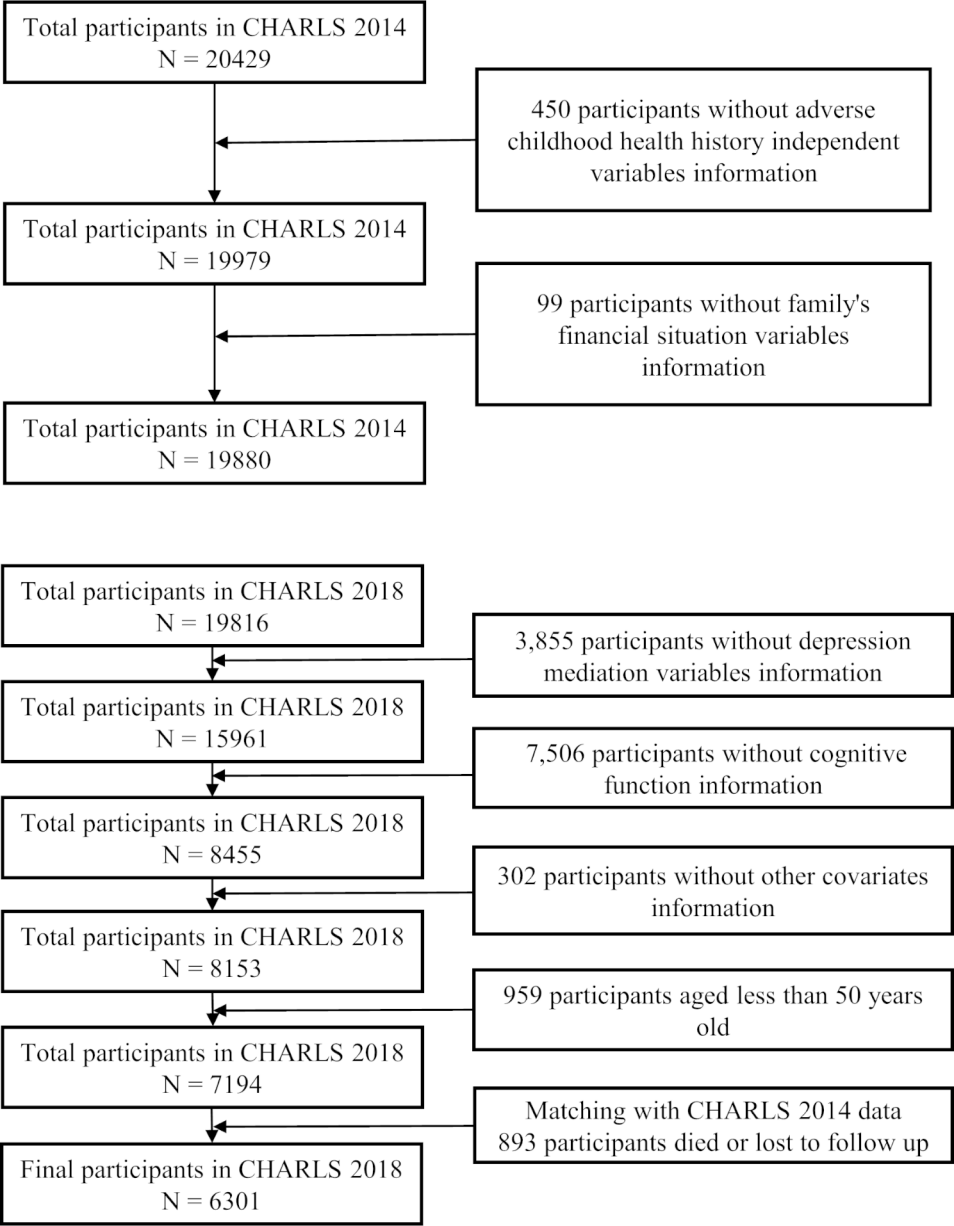
Sample Selection Process

This study utilized a cross-sectional design, and the data were primarily collected from the China Health and Retirement Longitudinal Study (CHARLS) that covered the years 2014 and 2018. CHARLS is a nationally representative survey of individuals aged 45 and older in China. In 2011, a baseline survey was conducted. Following that, three follow-up surveys were conducted in 2013, 2015, and 2018. There was a collection of information on middle-aged and older adults for demographic and lifestyle variables, and a life history interview was conducted in 2014 in order to gather data regarding childhood health experiences. Although many studies have focused on MCI in older adults [54–56], there are studies indicating that midlife represents the critical stage when the brain begins its transition to aging [57, 58], and the study on cognitive decline in the middle-aged and elderly population has become a research priority [59]. Therefore, it is acceptable to cover the middle-aged and elderly population in the study of cognitive function. Finally, there were 6301 middle-aged and older adults over the age of 50 in the total sample that were retained after missing values for relevant variables were excluded. The process of sample screening is shown in Fig. 2.

### Variable

#### Cognitive function

As part of the study, the cognitive function of middle-aged and older adults was considered the dependent variable. There are several methods of measuring cognitive function in individuals in the literature, situational memory capacity and mental status are commonly used as a measure of cognitive function [60, 61].

The concept of situational memory refers to the ability to recall specific events in a given situation. As part of the CHARLS questionnaire, the interviewer read ten words to the respondent and asked the participants to recall the words they heard three different times, and each word recalled was given a score of 1. It was calculated that the situational memory score, which ranged from 0 to 10, would be based on the average of the three recall scores.

Mental status was measured by the number of correct answers to five mathematical questions, whether the participants knew the year, month, day, week, and season at the time of the interview, and the ability of participants to redraw the picture. For every correct answer, 1 point would be given, and the total score was added to the mental state score, which was on a scale of 0 to 11, based on the number of correct answers. After that, a cognitive function score was computed by adding the situational memory and mental state scores, where the cognitive function score ranged from 0 to 21.

#### Adverse Childhood Health Experiences

Based on self-reports, health experiences during childhood (before the age of 15) were assessed among middle-aged and older adults over 50 years of age. In accordance with the CHARLS Life Interview Questionnaire, the items of the ACHEs in our study included: the health status compared to other children of the same age (ACHE1), missing school (ACHE2), being confined to bed (ACHE3), and length and frequency of hospitalizations (ACHE4 & ACHE5).

In order to represent the exposure level of ACHEs, we recoded ACHE1, about average and healthier were assigned a value of 0 [62]. The final results of the recoding are as follows: About average and Healthier = 0, Somewhat less healthy = 1, Much less healthy = 2. In addition, the score increased by 1 point if each of another four items from ACHEs occurred. After the calculation was done, the score could be used to determine the degree that the adult had been exposed to ACHEs. The higher score means the more severe ACHEs. In this study, the total values ranged from 0 to 6.

#### Depression

Depression was measured by using a revised 10-item version of the Center for Epidemiologic Studies Depression Scale (CES-D), which has been adapted in the CHARLS questionnaire [63]. A four-point scale was used to rate eight negative statements (e.g., I feel depressed) and two positive statements (e.g., I am happy). By reverse-scoring the two positive statements, we calculated the scores of the ten items, the higher the score, the more severe the depression. The 10-item CES-D scale was validated in a study conducted by Chen and Mui with elderly Chinese respondents [64]. And the CES-D-10 used in this study demonstrated reasonable internal consistency with α = 0.8049.

#### Covariates

In this study, there were three aspects of covariates, including demographics, health status and functioning, and socioeconomic conditions. Age (50 ~ 60 years old and 60 ~ 95 years old), gender (male and female), marriage (unmarried and married), education, and place of residence were included in the demographics; residences were categorized based on the CHARLS questionnaire options as Central of City/Town = 1, Urban-Rural Integration Zone = 2, Rural = 3, and Special Zone = 4, and education was coded as Illiterate = 0, Elementary School = 1, Middle School = 2, High School = 3, College or above = 4.

Aside from chronic disease conditions (yes and no), sleep status, smoking and drinking habits, and activities of daily living (ADL) were included in health status and functioning. As part of the CHARLS questionnaire, respondents were asked to specify six activities of daily living (ADL) that they perform on a daily basis, which included dressing, bathing, eating, transferring, incontinence, and toileting [65]. ADL was treated as a dichotomous variable. In the event that any difficulty was reported, the reporter would be recognized as having difficulty with ADL [66]. Accordingly, we coded 0 for respondents with difficulty with ADL and 1 for respondents without such difficulties. In accordance with the Healthy China Initiative (2019–2030), sleeping more than seven hours at night was defined as enough sleep, whilst not enough sleep was defined as spending at least seven hours [67].

With the questionnaire, we assessed smoking and drinking status not only in the last 12 months but also in the questions “Do you still have the smoking habit or have you totally quit?” and “When did you quit or reduce drinking?”. As part of our approach to estimating smoking and drinking covariates, we took into account quitting smoking and drinking as well as having a history of smoking and drinking. Finally, smoking and drinking habits were coded as dichotomous variables, where yes and no represent the presence or absence of a history of smoking and drinking.

According to fundamental cause theory (FCT), higher socio-economic status (SES) is associated with better health outcomes through health-promoting behaviors [68, 69], and there was evidence that indicated that health behaviors may be influenced differently by the economics and education of the SES [70]. In order to measure SES, variables such as the education of parents, the economic status of childhood families, and individual income were considered.

The question on the economic status of childhood families in the CHARLS 2014 questionnaire was as follows: “When you were a child before age 17, compared to the average family community/ village at that time, how was your family’s financial situation”, the question had four options, which were 1 = a lot better off than them; 2 = somewhat better off than them; 3 = same as them; 4 = somewhat worse off than them; 5 = a lot worse off than them. Accordingly, we recoded worse as 0 for poor childhood family financial circumstances, similar to them as 1 for moderate childhood family financial circumstances, and better as 2 for good childhood family financial circumstances. Further, as a result of the complexity of the CHARLS income item and the fact that elderly individuals do not have fixed incomes, the final sum of individual income includes negative cases. Thus, we used household per capita expenditure (PCE) as a proxy for household per capita income and logarithmize it. The coding of the variables is shown in Table 1.

**Table 1:** Coding of variables

Variable	Indicator Description and Assignment
**Age**	50 ~ 60 years old = 0, 60 ~ 95 years old = 1
**Gender**	Female = 0, Male = 1
**Residence**	Central of City/Town = 1, Urban-Rural Integration Zone = 2, Rural = 3, Special Zone = 4
**Education**	Illiterate = 0, Elementary School = 1, Middle School = 2, High School = 3, College or above = 4
**Marital status**	Unmarried = 0, Married = 1
**Drink**	No = 0, Yes = 1
**Smoke**	No = 0, Yes = 1
**Sleep situation**	Enough sleep (≥ 7 h/day) = 1, Non-enough sleep (< 7 h/day) = 0
**ADL**	Impaired = 0, Non-Impaired = 1
**Chronic**	No = 0, Yes = 1
**ChildSES**	Worse = 0, Same as them = 1, Better = 2
**Paternal education level**	Illiterate = 0, Non-illiterate = 1
**Mother’s education level**	Illiterate = 0, Non-illiterate = 1
**PCE**	log of per capita expenditure
**Depression**	0 ~ 30
**ACHEs**	0 ~ 6
**ACHE1 (Health Status)**	About average or healthier = 0, Somewhat less healthy = 1, Much less healthy = 2
**ACHE2 (Missing School)**	No = 0, Yes = 1
**ACHE3 (Confined to Bed)**	No = 0, Yes = 1
**ACHE4 (Hospitalized for a month or longer)**	No = 0, Yes = 1
**ACHE5 (Hospitalized more than three times)**	No = 0, Yes = 1
**Cognition**	0 ~ 21

#### Statistical analysis

Firstly, we used the mean ± standard deviation of continuous variables for describing the basic characteristics of participants, as well as proportions for categorical variables. Additionally, Judd and Kenny’s suggestions [71], as well as Baron and Kenny’s causal steps approach [72] were used to explore the relationship between adverse childhood health experiences (ACHEs), depression, and cognitive function.

**Fig. 3:**
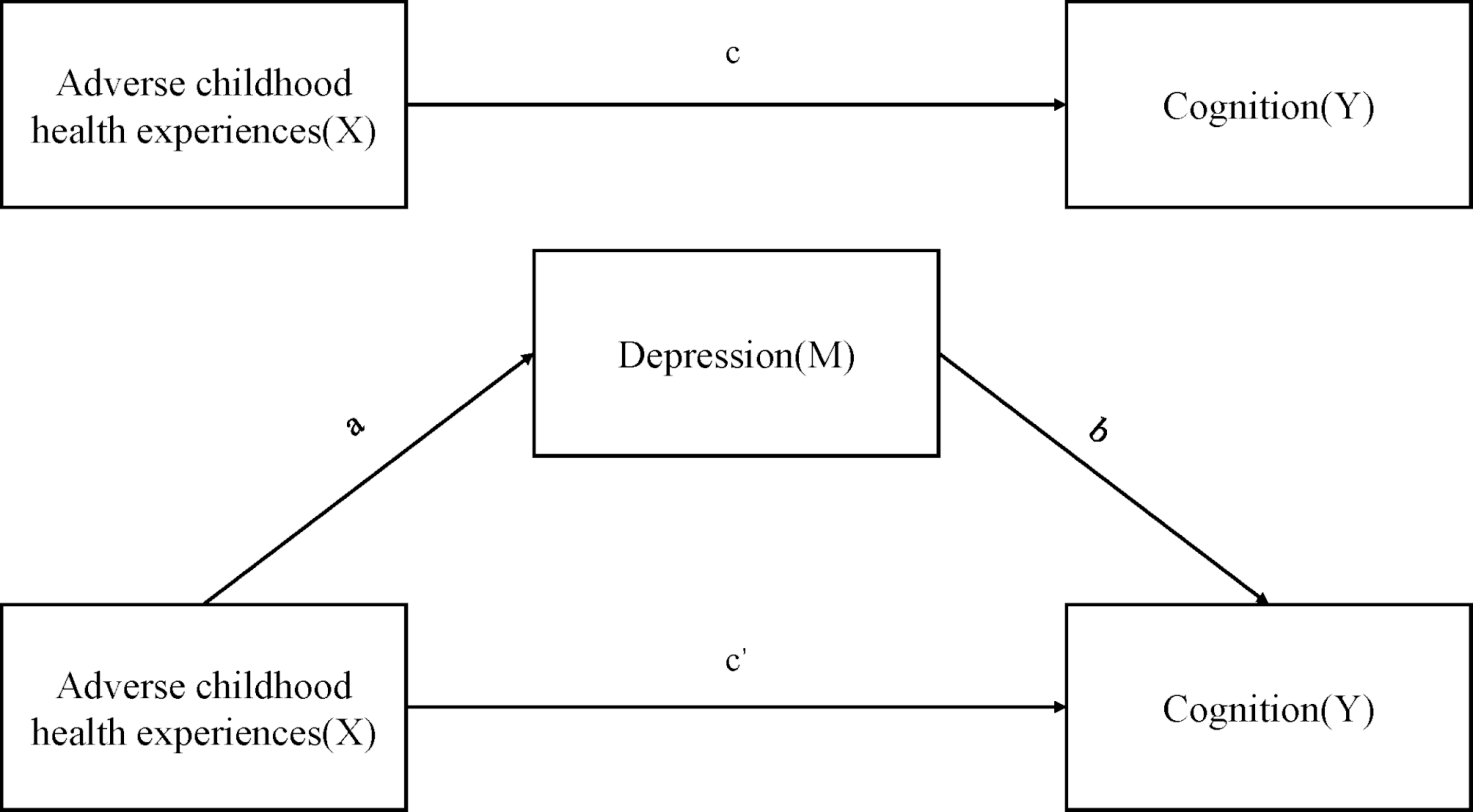
Research Pathway Hypothesis

As a result, the following three regression processes would be estimated, thereby validating the propagation of mediated effects:

They are first regressing the independent variable (X) on the mediating variable (M) to verify whether it is correlated (Fig. 3, path a). Second, a regression is carried out between the independent variable (X) and the dependent variable (Y) in order to verify the relevance of the relationship (Fig. 3, path c). Third, both the independent variable (X) and the mediating variable (M) will be regressed on the dependent variable (Y) to verify whether the mediating variable (M) is correlated with the dependent variable (Y) and also to test whether the effect of the independent variable (X) on the dependent variable (Y) is diminished by mediating variable (M) (Fig. 3, path b & path c’). In this case, it is regarded as fully mediated when the final effect of the independent variable (X) on the dependent variable (Y) is no longer significant.

It was our intention to test the mediating effects in the empirical evidence through the Sgmediation command in Stata software, as well as the significance tests used to analyze these mediating effects, which included the Sobel test [73], the Goodman test 1, and the Goodman test 2.

It is pertinent to note that the dependent variable in this study was the cognitive function of middle-aged and elderly people. As a continuous variable, it is possible to evaluate the cognitive ability of the sample, and using it as a categorical variable enabled us to determine whether the cognitive function of the sample was satisfactory or not.

Therefore, two methods of analysis would be employed in this study. Based on a generalized least squares regression model, we validated the mediating role of depression in the relationship between adverse childhood health experiences (ACHEs) and cognitive functioning. Then we used a logistic regression model based on the KHB method [74, 75] for robustness testing. STATA16 was used for the statistical analysis of all procedures.

## Results

### Description statistics

According to the results of descriptive statistics, the mean score of cognition is 13.79 (± 3.07). The characteristics of the participants are shown in Table 2. Among the 6301 respondents, more adults (55.69%) were over 60 years of age. Males constituted 57.18% of the sample, a substantial proportion of people live in rural areas (67.56%), and the majority of participants were elementary school educated (43.50%), married (89.95%), non-impaired in activities of daily living (50.94%), and had no history of smoking (51.94%) and drinking (55.64%). Further, compared with the father, the mother had a higher proportion of illiteracy (79.69%) when it comes to education level. In addition, the mean scores of PCE were 6.23 (± 0.94), the mean scores of depression were 7.37 (± 5.94), and the means of ACHEs were 0.29 (± 0.74).

**Table 2:** Characteristics of participants from CHARLS

Characteristics	N / Mean ± SD	%
**Age**		
50–60 years old	2792	44.31
60–95 years old	3509	55.69
**Gender**		
Male	3603	57.18
Female	2698	42.82
**Residence**		
Central of City/Town	1456	23.11
Urban-Rural Integration Zone	556	8.82
Rural	4257	67.56
Special Zone	32	0.51
**Education**		
Illiterate	305	4.84
Elementary School	2741	43.50
Middle School	1991	31.60
High School	1089	17.28
College or above	175	2.78
**Marital status**		
Married	5668	89.95
Unmarried	633	10.05
**Drink**		
Yes	2795	44.36
No	3506	55.64
**Smoke**		
Yes	3028	48.06
No	3273	51.94
**Sleep**		
0–7 h/day	3570	56.66
7–15 h/day	2731	43.34
**ADL**		
Impaired	3091	49.06
Non-Impaired	3210	50.94
**Chronic**		
Yes	2758	43.77
No	3543	56.23
**ChildSES**		
Worse	2281	36.20
Same as them	3291	52.23
Better	729	11.57
**Paternal education level**		
Illiterate	2891	45.88
Non-illiterate	3410	54.12
**Mother’s education level**		
Illiterate	5021	79.69
Non-illiterate	1280	20.31
**ACHE1**		
About average or healthier	5569	88.38
Somewhat less healthy	486	7.71
Much less healthy	246	3.90
**ACHE2**		
Yes	314	4.98
No	5987	95.02
**ACHE3**		
Yes	318	5.05
No	5983	94.95
**ACHE4**		
Yes	162	2.57
No	6139	97.43
**ACHE5**		
Yes	70	1.11
No	6231	98.89
**PCE, Mean ± SD**	6.23 ± 0.94	
**ACHEs, Mean ± SD**	0.29 ± 0.74	
**Depression, Mean ± SD**	7.37 ± 5.94	
**Cognition, Mean ± SD**	13.79 ± 3.07	
**Total**	6301	100

*Abbreviations: ACHEs = adverse childhood health experiences. ADL = activities of daily living. PCE = per capita expenditure. SD = Standard Deviation.*

Additionally, we provide additional information regarding the classification and continuum of adverse childhood health experiences (ACHEs) among middle-aged and older adults (*see Additional file* Table 1, *Additional file* Table 2, *and Additional file* Table 3). ACHE1 was observed in 732 (11.62%) middle-aged and older adults, whereas ACHE2 was observed in 314 (4.98%). The total number of individuals with ACHE3 was 318 (5.05%), 162 (2.57%) had ACHE4, and 70 (1.11%) had ACHE5. The proportion of male middle-aged and older adults who suffered from adverse childhood health experiences was higher than that of female middle-aged and older adults and the proportion of older adults (60 to 95) who had ACHE2, ACHE3, and ACHE4 was higher than that of middle-aged adults (50 to 60). It should also be noted that the proportion of children with elementary school education was generally higher than the proportion of children with other levels of education for all types of ACHEs. Meanwhile, the t-test results did not indicate a significant difference in ACHEs among individuals of different genders and educational levels.

### Relevance test

According to Baron and Kenny’s causal steps approach, we constructed basic regression models Model 1 ~ Model 3 in order to validate hypotheses for our research and to investigate the relationship between different types of ACHEs and cognitive function as well as depression. As shown in Table 3, our analysis of Model 1 demonstrated a positive relationship between all five types of ACHEs after controlling for three aspects of covariates. This suggested that middle-aged and older adults who had a variety of ACHEs were more likely to develop depression with increasing levels of these experiences. In Model 2, it was observed that cognitive functioning decreased when a person had ACHE3 in childhood. As a result, the cognitive functioning score was reduced by 0.385 units for each unit increase in ACHE3. And after adding the depression variable to Model 3, the relationship between ACHE3 and cognitive function ceased to be significant.

**Table 3:** Regression results of ACHEs and their items

Variable	Model 1	Model 2	Model 3
	Depression	Cognition	Cognition
ACHE1	1.100***	-0.106	-0.018
	(0.173)	(0.081)	(0.081)
ACHE2	1.381***	0.105	0.215
	(0.336)	(0.161)	(0.158)
**ACHE3**	1.861***	-0.385**	**-0.238**
	**(0.340)**	**(0.168)**	**(0.166)**
ACHE4	1.147**	-0.282	-0.191
	(0.464)	(0.210)	(0.210)
ACHE5	2.739***	-0.155	0.064
	(0.752)	(0.308)	(0.306)
**ACHEs**	0.817***	-0.082*	**-0.017**
	**(0.107)**	**(0.049)**	**(0.049)**

Standard errors in parentheses * p < 0.10, ** p < 0.05, *** p < 0.01; All control variables were included in the model

The negative effects of different adverse childhood health experiences (ACHEs) may interact with one another, thus affecting the development of children psychologically and cognitively for a longer period of time. In addition to affecting the development of children physically and mentally, the negative effects of different ACHEs may interact. Accordingly, we also present the results of the generalized least squares regression for ACHEs, after controlling for all relevant variables, the regression model showed that ACHEs were associated with depression at a significance level of 1% and had a significant negative impact on cognitive functioning at a level of significance of 10%. Each unit increased ACHEs in a 0.082-unit decrease in cognitive functioning. As a result of including the depression variable in Model 3, it is evident that ACHEs no longer had significant effects on cognitive function.

### Mediation effect analysis

As a result of our research, the mediating effect of depression was 36.92% for ACHE3, while 70.11% of the total effect of ACHEs was mediated by depression, as shown in Table 4, according to the Sobel mediating effect test.

**Table 4:** Results of the Sobel mediating effects test

	ACHE3	ACHEs
Sobel	-0.1471*** (Z= -5.27)	-0.0649*** (Z= -7.09)
Goodman-1 (Aroian)	-0.1471*** (Z= -5.26)	-0.0649*** (Z=-7.07)
Goodman-2	-0.1471*** (Z= -5.29)	-0.0649*** (Z=-7.10)
Indirect effect	-0.1471*** (Z= -5.27)	-0.0649*** (Z=-7.09)
Direct effect	-0.2512 (Z= -1.57)	-0.0276 (Z=-0.58)
Total effect	-0.3983** (Z= -2.47)	-0.0925* (Z=-1.92)
The proportion of total effect that is mediated	0.3692	0.7011

Standard errors in parentheses * p < 0.10, ** p < 0.05, *** p < 0.01; All control variables were included in the model

In the meantime, we present the results of Baron and Kenny’s causal stepwise method. Pathway a showed that ACHE3 (β = 1.8605, p = 0.000) was positively associated with depressive symptoms. Pathway b showed that depression was found to be associated with lower cognitive function (β=-0.0790, p = 0.000) after the inclusion of all covariates and ACHE3. Pathway c showed that ACHE3 in childhood had a negative impact on cognitive function (β=-0.3846, p = 0.022).

**Fig. 4:**
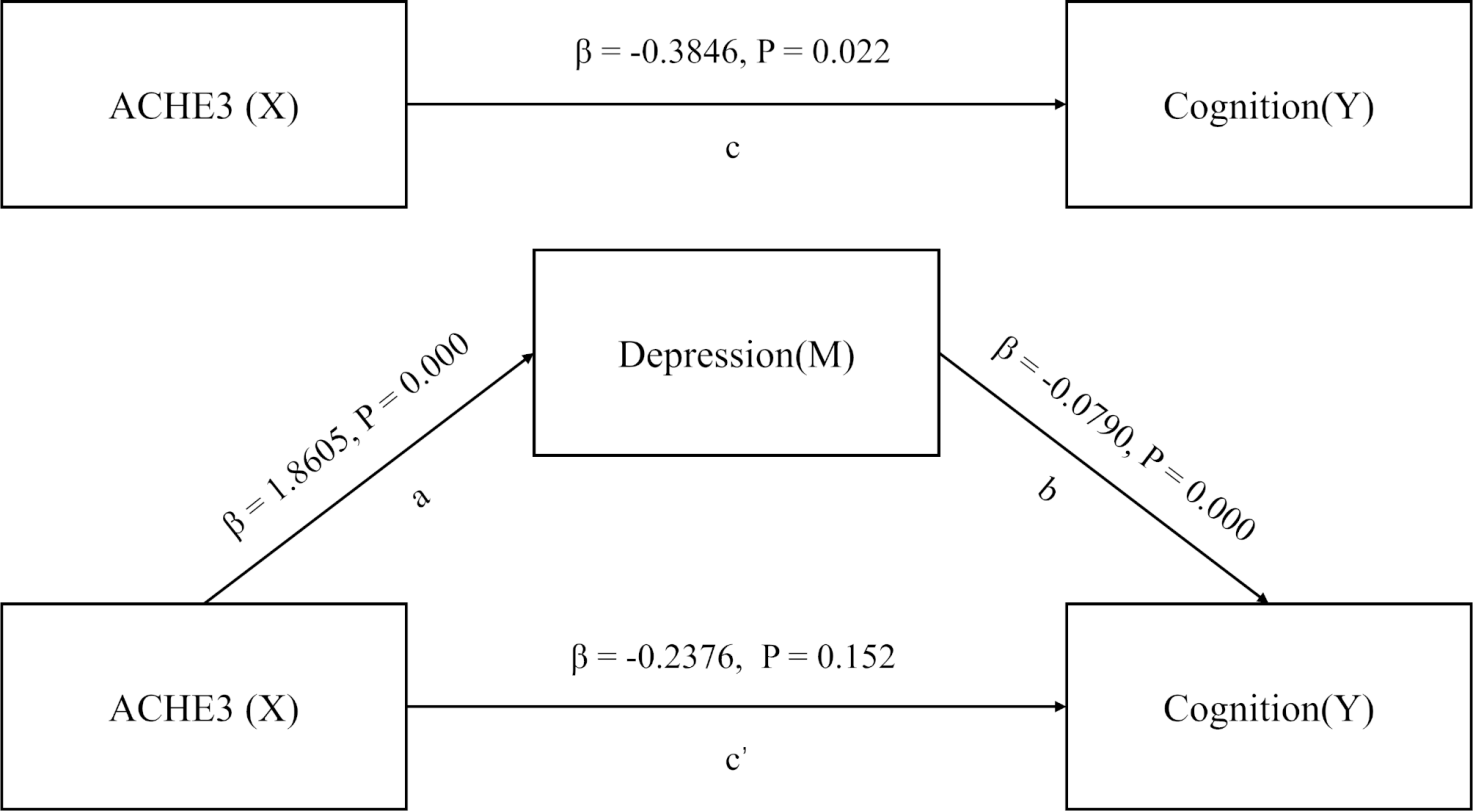
Depression mediates the relationship between ACHE3 and cognition

After introducing the variable of depression into the model, the regression coefficients of ACHE3 on cognitive function were no longer significant (β=-0.2376, p = 0.152). Accordingly, depression fully mediates the relationship between ACHE3 and cognitive functioning (Fig. 4).

**Fig. 5:**
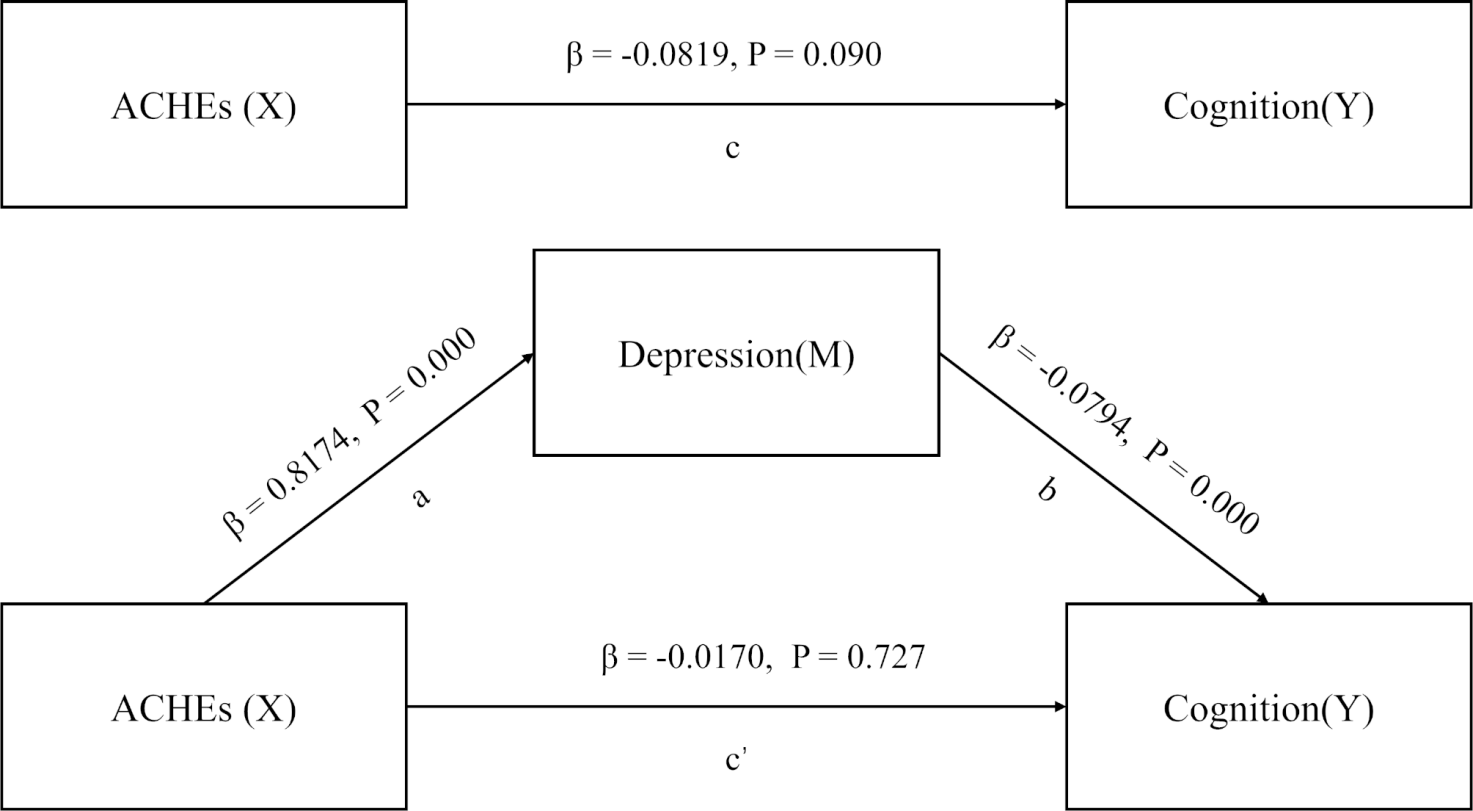
Depression mediates the relationship between ACHEs and cognition

Similarly, adverse childhood health experiences (ACHEs) were positively associated with depressive symptoms (β = 0.8174, p = 0.000). After adding all covariates and ACHEs, depression was found to be associated with lower cognitive functioning (β=-0.0790, p = 0.000) and ACHEs had a negative effect on cognitive function (β=-0.0819, p = 0.090). After introducing the variable depression into the model, the regression coefficient of ACHEs on cognitive function was no longer significant (β=-0.0170, p = 0.727). The relationship between ACHEs and cognitive functioning is fully mediated by depression as shown in Fig. 5. The findings confirmed the mechanism of the ACHEs on cognitive functioning, which is referred to “ACHEs - depression - cognitive function.”

### Heterogeneity analysis

Based on the previous results, we further explored the heterogeneity of the mediating role of depressive symptoms in the relationship between adverse childhood health experiences (ACHEs) and cognitive function across different groups. Among them, an examination of heterogeneity between gender groups was conducted using Model 4 ~ 6; an examination of heterogeneity between ages was conducted using Model 7 ~ 9; and an examination of heterogeneity between educational levels was conducted using Model 10 ~ 12. Tables 5 and 6, and Table 7 show some differences in this mediating role among gender, age, and education level.

When it comes to gender differences, ACHEs were more detrimental to females than to males, with an increase of 1.052 units in depressive symptoms and a decrease of 0.138 units in cognitive function for each unit increase in adverse childhood health experiences in middle-aged and older females. While the number of depressive symptoms increased by 0.651 units among middle-aged and older males who added ACHEs, cognitive function did not suffer a negative impact. As a result of including the variable of depressive symptoms in Model 6, ACHEs and cognitive functioning for female middle-aged and older adults were no longer correlated, suggesting that depression remained a mediating factor between ACHEs and cognitive functioning in female middle-aged and older adults.

**Table 5:** Results of the regression analysis of adverse childhood health experiences by gender

**Gender**	**Model 4**	**Model 5**	**Model 6**
	**Depression**	**Cognition**	**Cognition**
Male	0.651***	-0.045	0.007
	(0.116)	(0.062)	(0.061)
Female	1.052***	-0.138*	-0.057
	(0.159)	(0.077)	(0.077)

Standard errors in parentheses * p < 0.10, ** p < 0.05, *** p < 0.01; All control variables were included in the model

Across age groups, adverse childhood health experiences (ACHEs) were positively associated with depressive symptoms in middle-aged and older adults. But with increasing scores of ACHEs, older adults were negatively associated with reduced cognitive function, while middle-aged adults did not have a significant impact on cognitive function. The results of this study indicated that ACHEs were more likely to affect older adults compared to middle-aged individuals. When depression was included in Model 9, the same insignificant association between cognitive function and ACHEs emerged, suggesting that depression might also play a mediating role between ACHEs and cognitive function in older adults.

**Table 6:** Results of the regression analysis of adverse childhood health experiences by age

**Age**	**Model 7**	**Model 8**	**Model 9**
	**Depression**	**Cognition**	**Cognition**
50 ~ 60	0.815***	0.003	0.069
	(0.135)	(0.067)	(0.066)
60 ~ 95	0.809***	-0.149**	-0.086
	(0.133)	(0.069)	(0.069)

Standard errors in parentheses * p < 0.10, ** p < 0.05, *** p < 0.01; All control variables were included in the model

As far as levels of education were concerned, ACHEs were consistently and negatively associated with cognitive function among illiterate and college-educated and above populations. In contrast, ACHEs were not associated with cognitive function among middle-aged and older adults with elementary, middle, and high school education. Consequently, ACHEs have a more profound cognitive impact on illiterate and college-educated and above populations than on those with other levels of education. It was also found that ACHEs were positively associated with depression in people with different levels of education. The association between ACHEs and cognitive functioning remained negative when depression was added to Model 12 for the illiterate and college-educated and above populations, but the coefficient was reduced, suggesting that depression might have a partially mediating role between ACHEs and cognitive function in these two populations.

**Table 7:** Results of the regression analysis of adverse childhood health experiences by education

**Education**	**Model 10**	**Model 11**	**Model 12**
	**Depression**	**Cognition**	**Cognition**
Illiterate	0.865*	-0.613**	-0.551**
	(0.518)	(0.252)	(0.251)
Elementary School	0.649***	-0.090	-0.036
	(0.146)	(0.072)	(0.072)
Middle School	1.259***	-0.039	0.061
	(0.174)	(0.089)	(0.089)
High School	0.426**	0.151	0.175
	(0.210)	(0.110)	(0.109)
College or above	0.957**	-0.330*	-0.327*
	(0.379)	(0.192)	(0.196)

Standard errors in parentheses * p < 0.10, ** p < 0.05, *** p < 0.01; All control variables were included in the model

### Robustness analysis

Based on the KHB method, which decomposes the effects of discrete and continuous variables and extends the decomposability of the linear model, we tested the robustness of mediating effects by using a logistic regression model. Consequently, cognitive function was treated as binary variables, our study used the criteria of aging-associated cognitive decline (AACD) to Categorize cognition. The AACD is widely used by clinicians and scholars to diagnose mild cognitive decline [76]. At 5-year intervals, we divided the respondents into different age groups. It should be noted that the cognitive scores above SDs from the mean (age-appropriate norms) were coded as 1 which implies that cognition is considered to be non-impaired, whereas the cognitive scores below SDs from the mean were coded as 0 which implies that cognition is considered to be impaired. And a score of ≥ 10 on the CESD-10 was defined as presenting depressive symptoms, as suggested by Andreson [77].

According to Table 8, we report the results of the KHB approach for calculating the mediating effect. After re-adjusting for the classification of continuous variables, it was found that the indirect effect of ACHE3 on cognitive function was − 0.0504 (95% CI: -0.0788 ~ -0.0220). As for ACHEs, it showed that the indirect effect on cognitive function was − 0.0214 (95% CI: -0.0317 ~ -0.0111). It is evident that the mediating effect persisted even after adjusting for the type of variables.

**Table 8:** Total and direct health status and mediators on cognition

Effect	Variable	β	SE	Z	P	95% CI	
						Lower	Upper
**Indirect effect**	ACHE3	-0.0504	0.0145	-3.48	0.001	-0.0788	-0.0220
	ACHEs	-0.0214	0.0053	-4.07	0.000	-0.0317	-0.0111

According to the heterogeneity test, there was heterogeneity in regression coefficients between Model 5, Model 8, Model 10, Model 11, and Model 12. Therefore, we introduced an interaction between stratified variables and depression in order to verify it. The heterogeneity verification models Model 5a ~ Model 12a were constructed by adding the letter “a” to the original model names, respectively and the results are presented in Table 9. There was still the possibility of observing a significant coefficient after the interaction was introduced. This indicated that there was variation among groups in the construction of the models and therefore, the heterogeneity test results were still valid.

**Table 9:** The validation of heterogeneity

Variable	Model 5a	Model 8a	Model 10a	Model 11a	Model 12a
**Gender×Depression**	-0.069***				
	(0.009)				
**Age×Depression**		-0.074***			
		(0.008)			
**Education×Depression**			0.476***	-0.030***	-0.028***
			(0.004)	(0.004)	(0.007)

Standard errors in parentheses * p < 0.10, ** p < 0.05, *** p < 0.01; All variables were included in the model

## Discussion

We used cross-sectional data from the China Health and Retirement Longitudinal Study (CHARLS) of 2014 and 2018. It was found that adverse childhood health experiences (ACHEs) negatively correlated with cognitive function and that depression, according to mediation analysis, mediated the relationship between these two variables. As a result of the various types of adverse childhood health experiences, it is clear that being confined to bed (ACHE3) was negatively associated with cognitive function. Aside from that, based on different gender, age, and educational levels, the heterogeneity test indicated that the relationship between ACHEs and cognitive function and mediating effects of the depression were different.

According to our findings, the negative effects of ACHEs on cognitive function were consistent with those of previous studies. For example, a study on the 1983 Famine in Ghana, discovered that early childhood malnutrition was associated with significant, direct, and negative effects on cognitive development in adolescents and adults that persisted throughout their lives [78]. Similarly, another longitudinal study that involved over 6,100 older participants showed that early health adversity, such as being thinner than average at a young age, was associated with slower cognitive decline as time progressed [79]. Based on an analysis of the early life epidemiology of Alzheimer’s disease, it was also found that childhood physical illness negatively affected adult cognition and the structure of the brain as an adult [80].

There is no doubt that previous studies also supported the hypothesis that adverse childhood health experiences might contribute to depression. According to a study that was conducted on Chinese adolescents recently, childhood physical disabilities or long-term health problems had been shown to be associated with an increased risk of depression [81]. Furthermore, childhood trauma is known to be associated with adult depression, and it is believed that childhood trauma can be a risk factor in chronic depression as well [82–84]. From a neurobiological perspective, Yu et al. also found that the presence of a history of childhood trauma was associated with abnormal connectivity in the brains of adults with severe depression as a result of childhood trauma-related exposure to their environment [85]. As well previous studies had also connected poor childhood health with depressive symptoms. For example, Raposa et al. revealed that poor physical health in childhood might contribute to increased health-related stress and inadequate social function in adulthood, eventually leading to worsening depressive symptoms [86].

Depression has been shown to explain the mechanisms responsible for poor cognitive function and even dementia in some cases, and the significant differences in the relationship between depression and cognitive function due to age may be related to the reserve capacity theory of the brain. There is a definite correlation between depression and dementia in later life, which is a result of the brain reserve capacity theory first advocated by Satz, and the reserve threshold theory provides insight into the underlying mechanisms that may underlie the association of depression and dementia in later life [87]. A substantial amount of evidence suggested that depression damages neurons through a variety of pathways, leading to reductions in cognitive reserve capacity and increased emergence of impaired cognition sooner or more frequently under the influence of depression. Depression, for example, has been linked to vascular disease in the frontal striatal region, particularly in those suffering from depression [88, 89]. Furthermore, it is associated with increased levels of glucocorticoids [90], amyloid deposition, and neurogenic fiber formation [91], all of which may result in hippocampal damage and increased brain damage, reducing the cognitive reserve threshold of the brain and result in cognitive impairment. Furthermore, this could explain why pathways leading to poor cognitive function were expressed earlier in the elderly early life when compared with middle-aged adults, and to the extent that the effects were more pronounced among older adults.

Depression was found to mediate the relationship between ACHEs and cognitive functioning, which is consistent with findings relating to ACEs. Evidence suggests that depressive symptoms may be indirectly responsible for cognitive decline following ACEs [48, 92]. It indicates that adverse childhood health experiences might lead to depression which can increase the risk of cognitive impairment in the middle-aged and elderly population. Additionally, we found that the mediating role of depression between adverse childhood health experiences and cognitive functioning was different by gender. Similarly, middle-aged and older Chinese women were more likely to suffer from cognitive impairment in a recent study [62]. Differences brought by gender can be explained by emotions, which are a significant component of psychopathology [93]. These emotions encompass cognitive processes that are low in intensity but relatively long in duration [94, 95], which can lead to explanations of gender differences. It has been reported that female brains contain only half as much of the neurotransmitter (cerebral serotonin) that influences their emotional behavior as brains of male brains when compared to their male counterparts [96]. According to Nolen-Hoeksema, the increased vulnerability of females to depression is mainly related to gender differences in coping with an initial lowering of emotion [97].

Only limited research had been conducted on the differential effects of adverse childhood health experiences on cognitive functioning at the educational level. Most previous studies have found that education is an important factor in cognitive development [98, 99]. A study using data from the Survey of Aging and Retirement (SHARE) of individuals approximately 60 years of age suggested that education was associated with a protective effect against cognitive decline [100]. In our study, we found that ACHEs had a more profound effect on cognitive functioning and that depression might play a mediating role between them, in populations who were illiterate or college-educated and above.

The main advantage of this study is that the data were from a database of the national population survey, which provided abundant data to explore the mediating effect. It turned out that we did find a mediating effect of depression and further verified the statistical significance as well as the effect proportion of the mediating effect based on the Sobel and KHB method. In spite of this, our study has several limitations that need to be considered. First, a cross-sectional design was used since adverse childhood health experiences are not likely to change over time. So that we were still unable to determine the sequential relationship between the occurrence of depression and cognitive decline, which prevented us from establishing a causal relationship between them. Future longitudinal studies will be needed to determine the generalizability and causality of the findings. Second, due to the fact that we used database data, we only considered partial types of adverse childhood health experiences and covariates. Therefore, it is necessary to include more types of adverse childhood health experiences and covariates such as resilience to ACHEs and depression in the study in the future. Third, although numerous potential covariates were controlled, confounding factors may still exist and affect our findings.

## Conclusion

In conclusion, the results of our study demonstrated that the negative effects of adverse childhood health experiences on cognitive function were mediated through depression. For one thing, this emphasizes the significance of policy development that focuses on the physical condition of children and reduces their psychological stress. For another, it also indicates that improvements in cognitive function probably require effective intervention strategies during childhood to prevent the onset of dementia in middle and old age through interventions in risk factors associated with cognitive function so as to increase brain cognitive reserve thresholds early in childhood development and to establish a foundation for delayed cognitive decline in middle and old age.

## Electronic supplementary material

Below is the link to the electronic supplementary material.

Supplementary Material 1

## Abbreviations

ACHEs: Adverse Childhood Health Experiences
ACEs: Adverse Childhood Experiences
CHARLS: China Health and Retirement Longitudinal Study
KHB: The Karlson-Holm-Breen Method
MCI: Mild Cognitive Impairment
DALY: The disability-adjusted life year
CES-D: The Center for Epidemiologic Studies Depression Scale
ADL: Activities of Daily Living
SES: Socio-economic Status
PCE: Per capita expenditure
SD: Standard Deviation
