# Supplementary material for: Effects of adverse childhood health experiences on cognitive function in Chinese middle-aged and older adults: mediating role of depression
Source: BMC Public Health. 2023 Jul 5;23:1293. doi: 10.1186/s12889-023-16169-7 (PMC10320919; doi:10.1186/s12889-023-16169-7)
Supplement: Supplementary file 1 — Supplementary Material 1 [file 12889_2023_16169_MOESM1_ESM.docx]

**Additional file Table 1** Descriptive characteristics of the classification of adverse childhood health experiences (ACHEs) by gender and age.

| **Characteristics** | **Gender** | | $\boldsymbol{\chi}^{\boldsymbol{2}}$ |  | **Age** | |  |  |
| --- | --- | --- | --- | --- | --- | --- | --- | --- |
|  | **Male** | **Female** |  | P | **50 ~ 60** | **60 ~ 95** | $\boldsymbol{\chi}^{\boldsymbol{2}}$ | P |
|  | **(n = 3603)** | **(n = 2698)** |  |  | **(n = 2792)** | **(n = 3509)** |  |  |
| **ACHE1** |  |  | 2.4174 | 0.299 |  |  | 3.7356 | 0.154 |
| About average and healthier | 3204 (57.53) | 2365 (42.47) |  |  | 2470 (44.35) | 3099 (55.65) |  |  |
| Somewhat less healthy | 265 (54.53) | 221 (45.47) |  |  | 226 (46.50) | 260 (53.50) |  |  |
| Much less healthy | 134 (54.47) | 112 (45.53) |  |  | 96 (39.02) | 150 (60.98) |  |  |
| **ACHE2** |  |  | 6.2987 | 0.012 |  |  | 12.9399 | 0.000 |
| Yes | 201 (64.01) | 113 (35.99) |  |  | 170 (54.14) | 144 (45.86) |  |  |
| No | 3402 (56.82) | 2585 (43.18) |  |  | 2622 (43.79) | 3365 (56.21) |  |  |
| **ACHE3** |  |  | 6.6441 | 0.010 |  |  | 0.1134 | 0.736 |
| Yes | 204 (64.15) | 114 (35.85) |  |  | 138 (43.40) | 180 (56.60) |  |  |
| No | 3399 (56.81) | 2584 (43.19) |  |  | 2654 (44.36) | 3329 (55.64) |  |  |
| **ACHE4** |  |  | 7.8040 | 0.005 |  |  | 15.0578 | 0.000 |
| Yes | 110 (67.90) | 52 (32.10) |  |  | 96 (59.26) | 66 (40.74) |  |  |
| No | 3493 (56.90) | 2646 (43.10) |  |  | 2696 (43.92) | 3443 (56.08) |  |  |
| **ACHE5** |  |  | 0.0559 | 0.813 |  |  | 8.4059 | 0.004 |
| Yes | 41 (58.57) | 29 (41.43) |  |  | 43 (61.43) | 27 (38.57) |  |  |
| No | 3562 (57.17) | 2669 (42.83) |  |  | 2749 (44.12) | 3482 (55.88) |  |  |

**Additional file Table 2** Descriptive characteristics of the classification of adverse childhood health experiences (ACHEs) by education.

| **Characteristics** | **Edu** | | | | |  |  |
| --- | --- | --- | --- | --- | --- | --- | --- |
|  | **Illiterate** | **Elementary School** | **Middle School** | **High School** | **College or above** | $\boldsymbol{\chi}^{\boldsymbol{2}}$ | P |
|  | **(n = 305)** | **(n = 2741)** | **(n = 1991)** | **(n = 1089)** | **(n = 175)** |  |  |
| **ACHE1** |  |  |  |  |  | 38.53 | 0.00 |
| About average and healthier | 264(4.74) | 2403(43.15) | 1783(32.02) | 973(17.47) | 146(2.62) |  |  |
| Somewhat less healthy | 19(3.91) | 203(41.77) | 149(30.66) | 92(18.93) | 23(4.73) |  |  |
| Much less healthy | 22(8.87) | 135(54.44) | 59(23.79) | 24(9.68) | 8(3.23) |  |  |
| **ACHE2** |  |  |  |  |  | 12.90 | 0.01 |
| Yes | 3(0.96) | 137(43.63) | 104(33.12) | 57(18.15) | 13(4.14) |  |  |
| No | 302(5.04) | 2604(43.49) | 1887(31.52) | 1032(17.24) | 162(2.71) |  |  |
| **ACHE3** |  |  |  |  |  | 10.25 | 0.04 |
| Yes | 23(7.23) | 148(46.54) | 83(26.10) | 51(16.04) | 13(4.09) |  |  |
| No | 282(4.71) | 2593(43.34) | 1908(31.89) | 1038(17.35) | 162(2.71) |  |  |
| **ACHE4** |  |  |  |  |  | 6.45 | 0.17 |
| Yes | 5(3.09) | 69(42.59) | 45(27.78) | 35(21.60) | 8(4.94) |  |  |
| No | 300(4.89) | 2672(43.53) | 1946(31.70) | 1054(17.17) | 167(2.72) |  |  |
| **ACHE5** |  |  |  |  |  | 2.80 | 0.59 |
| Yes | 5(7.14) | 35(50.00) | 18(25.71) | 11(15.71) | 1(1.43) |  |  |
| No | 300(4.81) | 2706(43.43) | 1973(31.66) | 1078(17.30) | 174(2.79) |  |  |

**Additional file Table 3** Descriptive characteristics of adverse childhood health experiences (ACHEs) by gender, age, and education.

| **Characteristics** | | **ACHEs, Mean ± SD** | t | P |
| --- | --- | --- | --- | --- |
| **Gender** | Male（n = 3,603） | 0.30 ± 0.75 | -1.23 | 0.22 |
|  | Female（n = 2,698） | 0.28 ± 0.72 |  |  |
| **Age** | 50 ~ 60（n = 2,792） | 0.31 ± 0.77 | 1.68 | 0.09 |
|  | 60 ~ 95（n = 3,509） | 0.28 ± 0.71 |  |  |
| **Education** | Illiterate（n = 305） | 0.32 ±0.77 |  |  |
|  | Elementary School（n = 2,741） | 0.32 ±0.77 | 0.22 | 0.83 |
|  | Middle School（n = 1,991） | 0.26 ±0.68 | 1.52 | 0.13 |
|  | High School（n = 1,089） | 0.27 ±0.71 | 1.17 | 0.24 |
|  | College or above（n = 175） | 0.40 ±0.91 | -0.97 | 0.33 |
